# Spiritual Writings

**Published:** 1852-04

**Authors:** 


SPIRITUAL WRITINGS.

We some time ago alluded to the "spiritual knockings," by which the heads of so many weak people have been turned in Rochester, Boston, New York, and some other places, and gave what we have no doubt is the true explanation of the mystery. In our last number we alluded to a new phase of this superstition, which is exciting at this moment the most intense interest in the cities where the "knockings" are so well received. We propose now to resume the subject for the purpose of showing, that there is nothing new in this manifestation—that phenomena analogous to it have been exhibited by the disordered nervous system over and over again.

It is one of the characteristics of these superstitions that they flourish best in large cities and populous communities. They make comparatively but little progress in the country, and for this reason most of our readers are not likely ever to see much of them. Nevertheless, we can not believe that they would hold us excusable if we passed these things wholly without notice, forming as they do part of the medical history of the times.

We assume, then, that there is some reality in these "writings" which have been absurdly referred to a "spiritual" agency, just as we are compelled to admit some of the phenomena of mesmerism. We believe, for example, in the mesmeric sleep, in a state of complete coma co-existing with it, in the double consciousness, the somnambulism and sleep-talking, the exaltation of one or more of the senses, the remarkable development of muscular power, and some manifestations of a similar character. There can be no difficulty in admitting all this, because we witness things analogous to it in catalepsy, hysteria, natural somnambulism, and certain other perverted states of the nervous system. Upon the same ground it is, that we are prepared to credit some of the statements which we have read in regard to the "spiritual writings;" of course, we reject the supernatural agency as absurd and monstrous. There are persons who discredit the whole matter, and refuse to investigate it. They denounce it as a humbug, and imagine that this is sufficient to put it down.

We do not so regard the affair. That there is a vast deal of deception about it, a great deal of superstition and folly, a great deal that is disgusting and detestable, no one feels more than we do; but what is the reality upon which it rests? How much fact has it as a basis? What semblance of truth does it bear about it that gives it such currency and such influence over the popular mind? We make some extracts from the *Boston Medical and Surgical Journal*, for February 8th, 1852, which contain an answer to these questions, and, we think, explain the mysterious phenomena. The article from which we make our quotations was written by Dr. Samuel Taylor of Petersham, Mass., and gives the writer's experience in "spiritual writing." Dr. Taylor, it is proper to premise, is no believer in the superstition, but as there were among his friends "some very serious, honorable, and respectable men who practised spiritual writings," he was induced to make trial how far he might be made a "medium." Accordingly, in a company of gentlemen where the subject had been introduced, he says—

I took the pen, and as I had a few days before received a letter from Heath, informing me of the death of a nephew of mine, John Franklin Temple, I invoked his spirit, directing him, if he was present, to write "yes." Some oscillations of my pen immediately commenced, and in less than a minute my hand moved off and wrote in a firm, bold manner, "yes." Perceiving the effect, I asked permission to go round the counter, to a writing desk. At the desk I repeated my experiments at leisure. I asked the spirit to write his name, and the pen wrote, "Franklin Temple." It afterwards occurred to me, that although we always called him Franklin, yet his signature was J. F. Temple. Upon that reflection occurring to me, my hand immediately moved off and wrote, "J. F. Temple." I asked him if he was in heaven. My hand wrote immediately "yes." I asked him if there was such a place as hell; and immediately my hand wrote "yes." I then asked him if all men went to heaven; my hand wrote boldly "no." All this was done in the most firm and unmistakeable manner, without the least voluntary effort on my part, and with the firmest intent that my hand should remain passive. I know I did not make a voluntary motion, but let the so-called spirits have my hand to do what they pleased with it. I invoked the spirit of Benjamin Franklin to write his name, and the name was written in the manner I have described above. When the last letter was finished, my hand began to go down under the name, and I could not think what it was about, but its gyrations soon executed the flourish as seen in the fac simile of Franklin's autograph.

After I returned to my house, I repeated the experiments with the same results. I invoked the spirit of my father to write his name, and the writing performed as usual, and I am satisfied that the hand-writing was a fac simile of his; the peculiarly awkward J I recognized, upon reflection, was such as I have seen him make. When I have (so to speak) called up the spirit of my nephew, the name is always written in a plain fair hand, and in a style I never should have written it voluntarily, and always in the same style. I have had no opportunity, however, of comparing it with the signature of my nephew, and do not know whether they correspond.

In my experiments I learned, the same evening, that the forefinger of my right hand would operate more strongly than the pen: and here I will digress by saying I think this will always be the case. The spirit may be willed to make every letter in the same place, when writing with the finger. In this way the letters are made in great perfection, insomuch that if you have a doubt as to the letter intended to be made, the moving power will sweep to the right or left more distinctly, or even make the letter over again. What is peculiarly worthy of remark is, that the moving power in making an O throws round convulsively oftentimes twice; *always* twice if you wish it.

Experimenting in this way with my finger, I asked if the spirit of my father-in-law was present. The answer was immediate, "yes." I asked him to spell out his name. "Alanson Lincoln" was immediately spelled out. I asked him if he was in heaven. Answer "yes." I asked him if the religion in which he was educated was the best for mankind—the nearest the truth. As near as I can recollect, the names of the different religions were passing through my mind, together with doubts as to how they might be viewed by us in futurity. The answer to the question was, "no." I will here say that Mr. Lincoln, in his lifetime, was what is termed "Orthodox," and most strict in his principles, and more than usually devoted to his religion. I then asked what religion was nearest the truth; and "Roman Catholic" was written out.

Subsequently Dr. Taylor repeated his experiments and obtained the following significant results:

I then thought I would inquire of a spirit more about different religions. I asked which was the best religion, at the same time fixing my mind sternly on the word Protestant. My hand immediately wrote "Protestant." In the same manner, and by direction of the same *spirit*, my hand wrote "Methodist," "Unitarian," and I believe one or two others. I could not make my hand write "Mormon;" the idea was too ridiculous. By this time, what little belief I had that these phenomena were the work of spirits, was pretty essentially demolished, and I asked if this was the work of the spirits of the departed. The answer was "no." I asked if it was the work of the devil. The answer was "no." I asked if it was *detached vitalized electricity*. The answer was "yes."

Here is the testimony of a sensible man to the fact, that his hand moved without a consciousness of voluntary effort on his part—moved in a way that at first struck him with surprise, and that seemed to have something marvellous in it. We think his testimony is not to be rejected, and the more because, in the facts stated, he has furnished what, to our mind, is a satisfactory clue to the mystery. He wrote, without being conscious of it, what was at the moment the conviction of his mind. At first he wrote "Roman Catholic;" then "Protestant," "Methodist," &c; but could *not* write "Mormon," because "the idea was too ridiculous." He did not believe the impulse was supernatural, and consequently his hand would not write "yes," when he asked whether it was the work of "spirits," or "the devil;" but instantly wrote "yes," when he asked if it was "detached vitalized electricity." It was not a voluntary movement, and yet was suggested by the mind, the hand performing an act "the consciousness of which was not returned to the common sensorium."

The state of the nervous centres in which such actions are performed, is a singular and inexplicable one. We call it "hysterical," and it is certain that the condition can be most readily induced in hysterical persons; but we are still as far as ever from understanding what the peculiar condition is. But it is no new manifestation of nervous power. It is the same which Mesmer availed himself of to produce effects which astounded his contemporaries, and by which the mesmerists and biologists of our day perform their feats so marvellous to "the million." In this condition, the will seems to have lost its directive force—to have become annulled, or obedient to the will of another, and the muscles of the individual act from the impulse of sympathy.

A more remarkable example of this perverted condition of the nervous system was, perhaps, never afforded than by the "*Jerks,*" which accompanied the intense religious excitement that prevailed in Kentucky and Tennessee about the beginning of the present century; and as that vagary seems to us to throw much light upon the mania under consideration, we will quote from a late writer some passages descriptive of the strange phenomenon. We refer to the Rev. Dr. Davidson. In his interesting and valuable "*History of the Presbyterian Church in Kentucky,*" he has collected with much labor many curious and instructive details on this subject, which are not less interesting to the physician than the theologian.

The first occurrence of the "Jerks," was at a sacramental meeting in East Tennessee, "when several hundred of both sexes were seized with this strange and involuntary contortion." Dr. Davidson remarks—

From the universal testimony of those who have described these spasms, they appear to have been wholly involuntary. Thus they have been represented by McNemar in the passage just cited. This remark is applicable also to all other bodily exercises.* What demonstrates satisfactorily their involuntary nature is, not only that, as above stated, the twitches prevailed in spite of resistance, and even the more for attempts to suppress them; but that wicked men would be seized with them while sedulously guarding against an attack, and cursing every jerk when seized. Travellers on their journey, and laborers at their daily work, were also liable to them.†

Instances have been given of men concealing whips on their persons, with the intention of using them upon the subjects or advocates of these contortions, who have themselves, to their great surprise and horror, been suddenly seized in a similar manner, and their whips have been violently jerked out of their hands to a distance. A young man, the son of an

---

*Stuartss Rem. No. II. Bibl. Rep. vi. 343.

†Stuart's Rem. No. II.

elder, who was a tanner, feigned sickness one Sabbath morning, to avoid accompanying the family to a camp-meeting. He was left alone in bed, with none others in the house but a few black children. He lay some time, triumphing in the success of his stratagem, but afraid to rise too soon, lest some one might be accidentally lingering and detect him. As he lay quiet with his head covered, his thoughts were naturally directed to the camp-meeting, and fancy painted the assembled multitude, the public worship, and individuals falling into the usual spasmodic convulsions. All at once he found himself violently jerked out of bed, and dashed round the room and against the walls, in a manner altogether beyond his control. Recollecting that praying was said to be a good sedative on such occasions, he resorted to the experiment, and to his great satisfaction found it successful. He returned to bed quite relieved, but only to be again affected in the same way, and to be again quieted by the act of praying. He then dressed himself, and, to occupy his mind, went to the tanyard, and drawing a skin from the vat, prepared to unhair it. He rolled up his sleeves, and, grasping the knife, was about to commence the operation, when, instantaneously, the knife was flirted out of his hand, and he himself jerked backward over logs and against the fences, as before. Gaining relief by resorting to the former remedy, he ventured to resume his occupation, and again he was interrupted. But, finding his talisman losing its efficacy, he began now to be really alarmed, and, quitting the yard, he returned to his chamber, and betook himself to prayer in good earnest. In this condition, weeping and crying to God for mercy, he was found by the family on their return.

Our space will not allow us to make more than one other quotation. Dr. D. continues—

Another example of the involuntary nature of these motions is presented in the case of a lady and gentleman of some note in the fashionable world, who were attracted to the camp-meeting at Cane Ridge by mere curiosity. On the way they diverted themselves with a variety of jokes upon the poor deluded creatures who allowed themselves to roll screaming in the mud and crying for mercy, and sportively agreed that if either of them should fall, the other should remain and render suitable protection and assistance. They had not been long on the ground when, to the consternation of the gentleman, his gay companion suddenly dropped; whereupon, instead of fulfilling his promise, he fled at full speed. Flight, however, proved no preservative, for he had not gone 200 yards before he was seized in the same way, and measured his length on the ground; while a crowd flocked round him to witness his mortification, and offer prayers in his behalf.*

[*To be continued.*]

---

*Hist. of Meth. in the W. States, No. X, (Gosp. Her. ii. p. 200.)

